# The Impact of Thyroid Hormones on Cardiometabolic Risk in Children and Adolescents with Obesity, Overweight and Normal Body Mass Index (BMI): A One-Year Intervention Study

**DOI:** 10.3390/nu16162650

**Published:** 2024-08-11

**Authors:** Eleni Ramouzi, Konstantina Sveroni, Maria Manou, Christos Papagiannopoulos, Sofia-Maria Genitsaridi, Athanasia Tragomalou, Aikaterini Vourdoumpa, Diamanto Koutaki, George Paltoglou, Penio Kassari, Evangelia Charmandari

**Affiliations:** 1 Division of Endocrinology, Metabolism and Diabetes, First Department of Pediatrics, National and Kapodistrian University of Athens Medical School, ‘Aghia Sophia’ Children’s Hospital, 11527 Athens, Greece; eleni_ramouzi@hotmail.gr (E.R.); konsver@gmail.com (K.S.); mariamanou93@hotmail.com (M.M.); christospapajohn@gmail.com (C.P.); sgenitsaridi@gmail.com (S.-M.G.); nansymou@hotmail.com (A.T.); katvourdouba@gmail.com (A.V.); mk_madw@hotmail.com (D.K.); gpaltoglou@gmail.com (G.P.); peniokassari@gmail.com (P.K.); 2 Division of Endocrinology and Metabolism, Center of Clinical, Experimental Surgery and Translational Research, Biomedical Research Foundation of the Academy of Athens, 11527 Athens, Greece

**Keywords:** obesity, thyroid hormone, cardiometabolic risk factors, thyroid function

## Abstract

Thyroid hormones regulate metabolism and have a major impact in maintaining cardiovascular homeostasis. The purpose of our study was to examine the relation of thyrotropin (TSH) and thyroid hormones with cardiometabolic parameters in children and adolescents with obesity, overweight, and normal body mass index (BMI) before and after the implementation of a comprehensive, multidisciplinary, personalized, lifestyle intervention program for 1 year. One thousand three hundred and eleven (n = 1311) children and adolescents aged 2 to 18 years (mean age ± SD: 10.10 ± 2.92 years) were studied prospectively. Patients were categorized as having obesity (n = 727, 55.45%), overweight (n = 384, 29.29%) or normal BMI (n = 200, 15.26%) according to the International Obesity Task Force (IOTF) cutoff points. All patients received personalized guidance on diet, sleep, and physical activity at regular intervals throughout the 1-year period. Detailed clinical evaluation and hematologic, biochemical and endocrinologic investigations were performed at the beginning and the end of the study. Subjects with obesity had a more adverse cardiometabolic risk profile than subjects with overweight and normal BMI on both assessments. At initial evaluation, total T3 concentrations were positively associated with uric acid and HbA1C, and free T4 concentrations were negatively associated with insulin concentrations, while there was no association between TSH concentrations and cardiometabolic risk parameters. Following the 1 year of the multidisciplinary, lifestyle intervention program, the concentrations of lipids, HbA1C, ALT, and γGT improved significantly in all subjects. Changes in TSH concentrations were positively associated with changes in systolic blood pressure (SBP), glucose, triglycerides, and cholesterol concentrations. Changes in free T4 concentrations were negatively associated with changes in cholesterol and insulin concentrations. Furthermore, changes in T3 concentrations were positively associated with changes in HbA1C, glucose, uric acid, and triglyceride concentrations. These findings indicate that in children and adolescents with overweight and obesity, thyroid hormones are associated with indices conferring cardiometabolic risk.

## 1. Introduction

Obesity in childhood and adolescence represents one of the most significant public health challenges of the 21st century. According to the World Health Organization (WHO), the prevalence of obesity nearly tripled between 1975 and 2016. In 2016, more than 1.9 billion adults were overweight, with over 650 million classified as having obesity. Concurrently, more than 340 million children and adolescents aged 5–19 years had overweight or obesity [1]. In Greece, the prevalence of overweight and obesity is approximately 21% among preschool aged children and up to 41% among older children and adolescents [2]. Compared with their normal-body mass index (BMI) counterparts, children and adolescents with obesity have a significantly higher risk of developing cardiometabolic complications and chronic diseases, such as insulin resistance, diabetes mellitus type 2 (DM2), hypertension, dyslipidemia, early onset atherosclerotic cardiovascular disease, non-alcoholic fatty liver disease (NAFLD), obstructive sleep apnea, and malignancies [3]. Given that obesity is associated with a wide range of comorbidities, early identification and intervention in modifiable risk factors is essential for preventing the onset of future health complications [4]. In our country, we developed innovative e-Health applications to address childhood obesity and showed that a multidisciplinary intervention program offering personalized advice on diet, sleep, and physical activity successfully reduced the prevalence of overweight and obesity in childhood and adolescence [5,6,7].

Thyroid hormones regulate metabolism and play a significant role in cardiovascular homeostasis [8,9,10]. The secretion of thyroid-stimulating hormone (TSH) is notably sensitive to the thyroid hormone concentrations and is frequently used clinically as a marker of their secretion [11]. TSH concentrations can vary within the same individual due to factors like circadian rhythm, genetics, or iodine consumption [12,13,14,15]. Elevated TSH concentrations, even within the normal range, have been associated with cardiovascular risk factors, such as BMI, lipid concentrations, and arterial blood pressure in adults, with some studies associating high-normal TSH concentrations with the development of metabolic syndrome [16,17,18,19]. Free thyroxine (FT4) has been negatively correlated with metabolic syndrome, while higher concentrations of free triiodothyronine (FT3) have been associated with increased waist circumference and BMI [20,21]. 

Recent studies have focused on the potential role of TSH, T3 and FT4 in the pathogenesis of cardiovascular disease (CVD) among children and adolescents with overweight and obesity. However, previous studies examining their association present inconclusive findings. In a retrospective study of children aged 2–18 years, TSH concentrations showed a positive correlation with BMI, triglyceride concentrations, fasting insulin, and insulin resistance as assessed by the Homeostasis Model Assessment for Insulin Resistance (HOMA-IR) index. Conversely, FT4 concentrations demonstrated a negative correlation with triglyceride concentrations [22]. In a separate study conducted in children and adolescents in Germany, TSH was positively associated not only with blood pressure, but also with total cholesterol, low-density lipoprotein cholesterol (LDL), and triglyceride concentrations. The strongest association between elevated lipid concentrations and TSH was observed in children who had overweight or obesity [23,24]. In addition, Radetti et al. reported that higher TSH concentrations were associated with increased total cholesterol and elevated blood pressure [25]. Meanwhile, Lundbäck et al. emphasized the positive association between TSH and fasting insulin, total cholesterol, and triglycerides [26]. Children and adolescents with obesity demonstrated significantly increased TSH and FT3 concentrations compared to those with normal BMI, with no corresponding increase in FT4 concentrations [27].

Furthermore, several studies have demonstrated that following weight loss, the thyroid dysfunction is restored and the concentrations of TSH, FT4, and T3 return to normal range [28]. However, other studies did not identify any associations between changes in TSH and thyroid hormone concentrations and alterations in BMI following lifestyle intervention [29,30]. The data regarding associations between changes in the concentrations of TSH, FT4, and T3 and changes in cardiovascular risk parameters are scarce [29,31,32]. Consequently, the purpose of the present study was to assess the relation of TSH and thyroid hormones with cardiometabolic parameters in children and adolescents with obesity, overweight, and normal BMI before and after the implementation of a comprehensive, multidisciplinary, personalized, lifestyle intervention program for 1 year. Focusing on children and adolescents is crucial for understanding the interplay between thyroid function and cardiometabolic risk due to rapid growth and hormonal changes that have an impact on metabolic processes. The early detection of cardiometabolic risk factors through thyroid function assessment allows for prompt intervention, which may help prevent the onset of chronic diseases in adulthood. Through a thorough examination of these associations, we also aim to contribute to the development of more focused interventions that comprehensively address the complex challenges presented by childhood obesity.

## 2. Materials and Methods

### 2.1. Study Design

This was a prospective observational cohort study of patients attending our Center for the Prevention and Management of Overweight and Obesity in Childhood and Adolescence, Division of Pediatric Endocrinology, Metabolism and Diabetes, First Department of Pediatrics, National and Kapodistrian University of Athens Medical School, ‘Aghia Sophia’ Children’s Hospital, who underwent a 12-month systematic, comprehensive and personalized lifestyle modification plan involving healthy diet, good quality sleep, and regular physical exercise [6]. Eligibility criteria included children and adolescents presenting for the evaluation and treatment of overweight or obesity. Participants were categorized into three BMI groups—obesity, overweight, and normal BMI—according to the International Obesity Task Force (IOTF) cut-off points. The classification process involved measuring each participant’s weight and height with precision instruments. Additional clinical parameters were assessed to ensure accurate categorization, including age, sex, and growth percentiles. This research study was carried out in compliance with the Helsinki Declaration and was approved by the Ethics Committee of Human Research of the ‘Aghia Sophia’ Children’s Hospital (Approval Number: EB-PASCH-MoM: 28/11/2013, Re: 10290-14/05/2013). The parents or legal guardians of the participants were given detailed information on the study’s objectives and methods and gave informed written consent, while subjects older than 7 years provided verbal consent prior to participation into the study.

### 2.2. Subjects

One thousand three hundred and eleven (n = 1311) children and adolescents (618 boys and 693 girls) aged 2–18 years were followed up for 1 year with planned appointments in accordance with our standard follow-up protocol. Subjects were categorized as having obesity (n = 727, 55.45%), overweight (n = 384, 29.29%), or normal BMI (n = 200, 15.26%) according to the IOTF cut-off points. All participants were assessed at frequent intervals by a multidisciplinary team that included a Pediatrician, Pediatric Endocrinologist, Pediatric Dietician, a professional fitness Personal Trainer and—when necessary—a Pediatric Clinical Psychologist. All subjects entered a personalized, multidisciplinary, lifestyle intervention program, which offered guidance and assistance to both participants and their families concerning healthy dietary choices, sleep, and exercise [5,6,33]. Children and adolescents with history of thyroid disease, positive antithyroid antibodies, or history of treatment for thyroid disease were excluded from the study.

### 2.3. Methods

#### 2.3.1. Anthropometric and Body Composition Parameters

One trained observer thoroughly obtained a detailed medical history and performed the clinical examination. Participants’ body weight was measured using a scale (Seca GmbH & Co. KG., Hamburg, Germany) while wearing light clothing and no shoes. Standing height was also measured without shoes using a Harpenden’s stadiometer (Holtain Limited, Crymych, Dyfed, UK). BMI was calculated as body weight (in kg) divided by height (in m) squared and expressed in kg/m^2^. Waist circumference (WC) was measured in standing position, following the WHO STEPS protocol, using a stretch-resistant tape (Seca GmbH & Co. KG., Hamburg, Germany). Measurements were taken horizontally midway between the lowest rib and the iliac crest at the end of a normal exhale. Hip circumference (HC) was measured at the widest point of the hips and buttocks. Blood pressure measurements, including systolic (SBP) and diastolic (DBP) blood pressure, were taken using a sphygmomanometer with an age-appropriate cuff (Comfort 20/40, Visomat, Parapharm, Metamorphosi, Attiki, Greece). In addition, every participant underwent bioelectrical impedance analysis (BIA) using the TANITA MC-780U Multi Frequency Segmental Body Composition Analyzer from Amsterdam, The Netherlands. This analysis aimed to estimate fat mass, fat-free mass, muscle mass, bone mass, total body water, and basal metabolic rate.

#### 2.3.2. Initial Assessment and Interventions

All participants arrived at the Endocrine Unit early in the morning on the study day. A detailed medical history and clinical examination were performed, including an assessment of pubertal status and standard measurements of weight, height, waist circumference, and hip circumference by a single trained observer. Blood samples were collected at 8:00 a.m. after a 12 h overnight fast for baseline hematologic, biochemical, and endocrinologic investigations. Samples were promptly centrifuged, separated, and stored at −80 °C until analysis [34,35]. Furthermore, body composition analysis was carried out.

Subsequently, all subjects were evaluated by a Pediatrician, Pediatric Endocrinologist and a Pediatric Dietitian for their daily nutritional habits. Using the USDA method, a 24 h recall of their meals was completed, detailing food and beverage intake, quantity, and timing. Questions were asked about usual eating habits and any specific dietary changes. This information was used to assess dietary patterns and nutritional status. Children and their parents received detailed information on the implications of obesity and the significance of adopting a healthier lifestyle as a family unit. Guidance was provided on modifying dietary habits, emphasizing the reduction in the consumption of processed food and the incorporation of fresh fruits, vegetables, whole grains, lean proteins, and healthy fats in line with the 2010 USDA guidelines and recommendations from the National Nutritional Guide for Infants, Children, and Adolescents. More specifically, personalized dietary recommendations involved three main meals (breakfast, lunch, and dinner) and two snacks (fruits, vegetables) during mid-morning and mid-afternoon. The goal was to develop a nutritious diet plan that matched the child’s food preferences, ensuring it was both enjoyable and feasible, while considering the food availability and preparation at school or at home. The significance of consuming breakfast was underscored, and the composition of the main meals was recommended based on “My Plate”, which illustrates the 2010 USDA guidelines [5,6,34,35,36].

In addition, detailed recommendations for adequate sleep were provided to each participant based on their age, according to the American Academy Consensus Guidelines. These guidelines suggested 9 to 12 h of sleep per day for children aged 10–12 years and 8 to 10 h per day for adolescents aged 13–18 years. Participants were advised to prioritize uninterrupted sleep, aiming to start sleeping as early as possible before midnight and maintaining a consistent sleep schedule each day [37]. Participants and their families were educated on the negative effects of insufficient sleep on metabolism and body weight. Also, children were advised to limit screen time to less than two hours per day, and to turn off all electronic devices one hour before bedtime.

Furthermore, a certified Personal Trainer assessed the physical activities and hobbies of children and adolescents on a weekly basis. His role included designing and implementing tailored exercise programs for children, providing guidance on physical activities and sports, motivating children to engage in regular exercise, and promoting the importance of leading an active lifestyle. The exercise plan involved participating in a chosen physical activity daily for 30 to 45 min, such as walking, jogging, cycling, and dancing [6].

Subjects with obesity were assessed monthly, subjects with overweight were assessed every two months, and those with normal BMI were monitored every three months. During each follow-up, anthropometric measurements were obtained, and a new 24 h diet recall was conducted, as previously described. Health professionals discussed the progress, adjusted goals, and encouraged the active involvement of parents and guardians in the process to provide additional support to children and adolescents. Detailed assessments of hematologic, biochemical, and endocrinologic parameters were carried out both at the beginning and the end of the study, as well as at 3–6 month intervals as required [6,34,35].

#### 2.3.3. Annual Assessment

Participants were admitted to the Endocrine Unit early in the morning for their annual assessment. A trained observer performed a comprehensive clinical examination, including Tanner staging and standard anthropometric measurements. These included weight, height, waist circumference, and hip circumference. Following these assessments, blood samples were collected at 08:00 h after a 12 h fast for detailed hematologic, biochemical, and endocrinologic analyses. In addition, body composition analysis was performed.

### 2.4. Assays

Hematologic analyses were conducted using the ADVIA 2110i analyzer from Roche Diagnostics GmbH, Mannheim, Germany. The ADVIA 1800 Siemens analyzer (Siemens Healthcare Diagnostics, Tarrytown, NY, USA) was utilized to measure glucose, total cholesterol, triglycerides (TG), and high-density lipoprotein cholesterol (HDL) concentrations. Apolipoprotein A1 (ApoA1), ApoB, and lipoprotein (a) [Lp(a)] concentrations were determined through means of latex particle-enhanced immunonephelometric assays using the BN ProSpecnephelometer by Dade Behring (Siemens Healthcare Diagnostics, Liederbach, Germany). Hemoglobin A1C (HbA1C) was quantified using an automated glycohemoglobin analyzer HA-8160 by Arkray (Kyoto, Japan) employing reversed-phase cation exchange high-performance liquid chromatography. Insulin resistance was evaluated with the homeostasis model assessment (HOMA-IR) method, calculated as follows: HOMA-IR = [fasting glucose (mg/dL) × fasting insulin (mU/L)]/405.

The concentrations of TSH, FT4, T3, antithyroid peroxidase antibodies, anti-thyroglobulin antibodies, adrenocorticotropin (ACTH), cortisol, insulin-like growth factor-I (IGF-I), and insulin-like growth factor binding protein-3 (IGFBP-3) were assessed using chemiluminescence immunoassays on the IMMULITE 2000 system from Siemens Healthcare Diagnostics Products Ltd. (Surrey, UK). Follicle-stimulating hormone (FSH), luteinizing hormone (LH), estradiol, ferritin, and insulin concentrations were measured with an automated electrochemiluminescence immunoassay analyzer (Cobas e411, Roche Diagnostics GmbH, Mannheim, Germany). Total 25-hydroxyvitamin D (25-OH-Vitamin D) concentration was determined using an automated electrochemiluminescence immunoassay on the Modular Analytics E170 analyzer (Roche Diagnostics, Basel, Switzerland).

### 2.5. Statistical Analysis

Results are shown as means ± standard deviation (SD) for continuous variables and as absolute and relative frequencies (%) for categorical variables. Anderson darling tests, histograms, and Q-Q plots were used to assess normality. At *p* < 0.05, statistical significance was established. ANOVA and Kruskal–Wallis tests were used to compare the distribution of continuous variables, normally distributed and not, respectively, among the three BMI groups (obesity, overweight, normal BMI). A paired-samples t test was used to compare the pre- and post-intervention results within each BMI category for variables that were normally distributed. Wilcoxon’s signed rank test was used to assess the relationships between skewed variables and participant groups. The Monte Carlo test or Pearson’s χ^2^ was used to investigate the relationships between categorical variables. When the conditions for the Pearson’s χ^2^ test were not met, Fisher’s χ^2^ test of independence (Fisher’s exact test) was used.

Subsequently, an investigation into the associations between thyroid hormones and cardiometabolic risk factors was conducted stratified by BMI (obesity, overweight, and normal BMI). Given the repeated measurements within the same subjects, a statistical modeling approach employing the Generalized Least Square (GLS) technique was applied to account for the correlation between the two time points. The initial modeling of variance was accomplished using the Restricted Maximum-Likelihood estimates (REML). REML served as a fitting method for linear mixed models, furnishing unbiased estimates of variance and covariance parameters. In the pursuit of models involving fixed effects that optimized variance modeling, a stepwise regression analysis was undertaken. The covariates used was fat mass, free fat mass, muscle mass, basal metabolic rate, glucose, cholesterol, insulin, uric acid, HDL, LDL, TG, SBP, DBP, HbA1C, creatinine, WC and HC, keeping each time age, gender, and pubertal status in the model. Variable selection was contingent on the fulfillment of a significance level of 0.157, as determined by maximum likelihood estimates. Model selection criteria, including the Akaike Information Criterion (AIC), Bayesian Information Criterion (BIC), and Likelihood-Ratio (LR) test, were employed to identify the most suitable models for the analysis. Imputation was performed on thyroid hormone variables, replacing missing values with their BMI-stratified median values. 

All statistical analyses were conducted using the R Project for Statistical Computing, version 4.3.1 (R Foundation for Statistical Computing, Vienna, Austria).

## 3. Results

### 3.1. Clinical Characteristics, Anthropometric, Hematologic, Biochemical, and Endocrinologic Parameters of All Subjects at Initial and Annual Assessment

One thousand three hundred and eleven (n = 1311) children and adolescents (618 boys and 693 girls) aged 2–18 years (mean age ± SD: 10.10 ± 2.92 years) were studied prospectively for one year. Subjects were classified as having obesity (n = 727, 55.45%), overweight (n = 384, 29.29%), or normal BMI (n = 200, 15.26%) according to the IOTF cutoff points. In Table 1, the clinical characteristics, hematologic, biochemical, and endocrinologic parameters of the participants are presented in all three BMI categories at the beginning and at the end of the study, 1 year after the implementation of the multidisciplinary, personalized, lifestyle intervention program. Children and adolescents with obesity had significantly greater WC, HC, and waist-to-hip ratio (WHR) in both assessments compared to children and adolescents with overweight and normal BMI. Following the implementation of the 1-year lifestyle intervention program, the percentage of participants with obesity decreased significantly by 15.1% (55.5% compared to 40.4%), while the percentages of participants with overweight and normal BMI showed a significant increase by 7% (36.3% compared to 29.3%) and 8% (23.3% compared to 15.3%), respectively. In addition, there was a significant reduction in the WHR in subjects with obesity and normal BMI (Table 1).

Furthermore, children and adolescents with obesity had significantly increased markers of inflammation [white blood cell count, erythrocyte sedimentation rate (ESR), ferritin], ALT, γGT, uric acid, and T3 concentrations, as well as lower concentrations of total 25-OH-Vitamin D compared to subjects with overweight and normal BMI. No significant differences in serum concentrations of both TSH and FT4 were noted among the three groups of participants at the initial and annual assessment (Table 1).

### 3.2. Cardiometabolic Risk Factors and Body Composition Parameters at Initial and Annual Assessment

Individuals with obesity had significantly higher SBP and DBP than those with overweight and normal BMI both at initial and annual assessment, while no significant reduction in SBP and DBP was noted following the 1-year lifestyle intervention program in the three groups. Regarding the lipid profile, individuals with obesity had higher concentrations of LDL and lower concentrations of HDL in both assessments compared to subjects with overweight and normal BMI. Furthermore, a significant increase in HDL and a significant decrease in LDL concentrations were noted following the lifestyle interventions in all individuals (Table 2). The concentrations of triglycerides and ApoB were significantly higher, while the ApoA1 concentrations were significantly lower in individuals with obesity than those with overweight and normal BMI in both assessments. The concentrations of fasting glucose and insulin, the HOMA-IR index, and HbA1C were significantly higher in individuals with obesity than the other two groups at both initial and follow-up assessment. The concentrations of total cholesterol and Lp(a) showed no significant difference between the three groups. 

Furthermore, after 1 year of the comprehensive lifestyle intervention program, individuals with obesity and overweight showed a notable reduction in body fat percentage. Meanwhile, all participants experienced significant increases in muscle mass percentage, bone mass, fat-free mass, total body water, and basal metabolic rate (BMR) (Table 2).

### 3.3. Cardiometabolic Risk Factors, Body Composition Parameters, and Thyroid Hormones at Initial and Annual Assessment: Comparisons According to Gender (Irrespective of Pubertal Status)

Supplemental Table S1 displays the cardiometabolic risk factors, thyroid hormones, and body composition measurements at both assessments according to gender, irrespective of pubertal status. Conversely, Supplemental Table S2 provides the same parameters categorized by pubertal status, regardless of gender. 

Both at initial and annual evaluation, there was no statistically significant difference in the concentrations of TSH, T3, and FT4 between boys and girls. In boys, there was a significant decrease in TSH, T3, and FT4 concentrations at the annual assessment compared with the initial assessment. In girls, there was a significant decrease in T3 and FT4 concentrations at the annual assessment compared with the baseline assessment. Regarding the cardiometabolic parameters, at initial assessment, cholesterol, LDL, ApoA1, glucose concentrations, HbA1C, and SBP were significantly higher in boys than girls, while at annual assessment, ApoA1 concentrations and SBP were higher in boys than girls. Furthermore, at both initial and annual assessment, fat percentage was significantly higher in girls than in boys, while the muscle mass, bone mass, free-fat mass, total body water, and basic metabolic rate (BMR) were significantly higher in boys than in girls. In both boys and girls, there was a significant decrease in fat percentage (*p* < 0.05) and a significant increase in free-fat mass (*p* < 0.05), muscle mass (*p* < 0.05), bone mass (*p* < 0.05), total body water (*p* < 0.05), and BMR (*p* < 0.05) at the annual assessment compared with the initial assessment (Supplemental Table S1). 

At both initial and annual evaluation, T3 and FT4 concentrations were significantly greater in prepubertal than in pubertal subjects, while there was no statistically significant difference in the concentrations of TSH between prepubertal and pubertal participants. In prepubertal subjects, there was a significant decrease in T3 and FT4 (*p* < 0.05) concentrations at the annual assessment compared with the baseline assessment. In pubertal subjects, there was a significant decrease in T3 (*p* < 0.05) and TSH (*p* < 0.05) concentrations at the annual assessment compared with the initial assessment. Regarding the cardiometabolic parameters, at both assessments, SBP (*p* < 0.05), DBP (*p* < 0.05), TG (*p* < 0.05), glucose, insulin concentrations (*p* < 0.05), and HOMA-IR (*p* < 0.05) were significantly higher in pubertal compared to prepubertal subjects, while cholesterol, LDL, HDL, ApoA1, and ApoB were significantly lower in pubertal than in prepubertal subjects. Furthermore, at both assessments, fat mass (*p* < 0.05), muscle mass (*p* < 0.05), bone mass (*p* < 0.05), free-fat mass (*p* < 0.05), total body water (*p* < 0.05), and basic metabolic rate (*p* < 0.05) were significantly higher in pubertal than in pre-pubertal subjects. In both prepubertal and pubertal subjects, there was a significant decrease in fat percentage (*p* < 0.05) at the annual assessment in comparison with the initial assessment (Supplemental Table S2). 

### 3.4. Mixed Regression Analysis of Thyreotropin and Thyroid Hormones Compared with Antropometric Parameters, Body Composition Parameters, Biochemical Parameters, and Cardiometabolic Risk Factors in All Subjects during the 1 Year of Lifestyle Intervention

As noted in Table 3, an increase in age by one year leads to a significant reduction in TSH, T3, and FT4 concentrations when the other variables remain constant. In addition, total T3 concentrations were positively associated with pubertal status (b = 4.258, *p* < 0.05), HbA1C (b = 9.107, *p* < 0.05), uric acid (b = 4.293, *p* < 0.05), and glucose concentrations (b = 0.338, *p* < 0.05) and negatively associated with creatinine concentrations (b = −64.537, *p* < 0.05). FT4 concentrations were associated positively with fat mass (b = 0.003, *p* < 0.05) and creatinine concentrations (b = 0.171, *p* < 0.05) and negatively with pubertal status (b = −0.043, *p* < 0.05) and insulin concentrations (b = −0.0005, *p* < 0.05). No correlations were noted between baseline TSH concentrations and cardiometabolic risk parameters.

### 3.5. Stepwise Regression Analysis of Thyrotropin, Thyroid Hormones and Anthropometric Parameters, Body Composition Parameters, Biochemical Parameters, and Cardiometabolic Risk Factors in All Subjects during the 1 Year of Lifestyle Intervention

BMR, FATM, puberty, age, gender, cholesterol, insulin, and creatinine concentrations were identified as significant predictors of FT4 concentrations. Among them, only FATM (b = 0.003) and creatinine (b = 0.198) were positively associated with FT4. However, BMI did not show a significant association with FT4 concentrations. Changes in glucose, cholesterol, triglycerides, and SBP showed significant positive associations with changes in TSH, suggesting that greater concentrations of these factors were associated with increased TSH concentrations. In addition, older age (b = −0.056) was associated with lower TSH concentrations. However, gender and BMI did not demonstrate a strong relation with TSH. Positive associations were observed between T3 concentrations and PMM, glucose, uric acid, TG, HbA1C, pubertal status, and obese subjects. Conversely, negative associations were found with FFM, HDL, creatinine, and age. These findings suggest that various cardiometabolic, biochemical and body composition factors play a significant role in influencing T3 concentrations, while gender may not have direct effects in this context (Table 4A–C).

### 3.6. Stepwise Regression Analysis among Changes in Thyreotropin, Thyroid Hormones and Anthropometric Parameters, Body Composition Parameters, Biochemical Parameters, and Cardiometabolic Risk Factors in Each BMI Category Separately during the 1 Year of Lifestyle Intervention

In subjects with obesity, FATM, FFM, puberty, age, gender, cholesterol, insulin and creatinine concentrations were identified as significant predictors of FT4 concentrations. Among them, only FATM (b = 0.002) and creatinine (b = 0.236) were positively associated with FT4 concentrations, while the rest of the parameters had a negative impact. Triglyceride concentrations (b = 0.004) and SBP (b = 0.012) showed significant positive associations with TSH, indicating that higher levels of these factors were associated with higher TSH concentrations. However, puberty and gender did not show a significant association with TSH. In addition, older age (b = −0.114) was associated with lower TSH concentrations. Positive associations were observed between T3 concentrations and glucose (b = 0.410), uric acid (b = 5.647), and TG (b = 0.051) concentrations. Conversely, negative associations were found with creatinine (b = −66.117) and age (b = −3.330). These findings suggest that various cardiometabolic and biochemical parameters may influence T3 concentrations, while gender and puberty may not have direct effects in this context (Table 5A–C).

The stepwise regression analysis in subjects with overweight showed the following: FATM, BMR, gender, cholesterol, TG and creatinine concentrations were identified as significant predictors of FT4 concentrations in subjects with overweight. Among them, only FATM (b = 0.005) and creatinine (b = 0.179) were positively associated with FT4 concentrations, while the rest of the parameters had a negative impact. However, puberty and age did not show a significant association with FT4. Cholesterol concentrations (b = 0.005) were positively associated with TSH concentrations, while puberty, age and gender did not show a significant association with TSH. Positive associations were observed between T3 and glucose (b = 0.331), uric acid (b = 4.221) concentrations, HbA1C (b = 11.922) and HC (b = 0.385). Conversely, negative associations were found with creatinine (b = −62.774), age (b = −2.242) and HDL concentrations (b = −0.224). These findings suggest that various cardiometabolic and biochemical parameters may influence T3 concentrations, while gender and puberty may not have direct effects in this context (Table 6A–C).

The stepwise regression analysis in normal-BMI subjects showed the following: Insulin concentrations (b = −0.004) and puberty (b = −0.108) were negatively associated with FT4 concentrations, while age and gender did not show a significant association with FT4. Insulin, cholesterol, and TG showed significant positive associations with TSH concentrations, indicating that higher concentrations of these parameters were associated with higher TSH concentrations. Positive associations were observed between T3 concentrations and insulin (b = 0.930), HbA1C (b = 14.050), and SBP (b = 0.367). Conversely, negative associations were found with HDL (b = −0.271), creatinine (b = −69.670), and age (b = −3.189). These findings suggest that various cardiometabolic and biochemical factors may influence TSH, T3 and FT4 concentrations, while puberty and gender may not have direct effects in this context (Table 7A–C).

## 4. Discussion

In this study, we investigated the associations between serum concentrations of TSH, FT4, and T3 and a wide range of cardiometabolic risk factors in euthyroid children and adolescents with obesity, overweight, and normal BMI at their initial assessment and 1 year after implementing a lifestyle intervention program on diet, sleep, and exercise. These associations were specific to the concentrations of T3 and FT4 and not evident for TSH concentrations. Changes in TSH, T3 and FT4 concentrations are also associated with changes in various cardiometabolic parameters in subjects with normal BMI, overweight and obesity, a fact indicating the involvement of thyroid hormones in the regulatory pathways of glucose and lipid metabolism. Moreover, the results of this study demonstrate that a multidisciplinary, personalized, lifestyle intervention program is effective in improving cardiometabolic risk factors, thereby preventing cardiovascular disease in children with overweight and obesity.

Regarding the cardiometabolic risk factors, children and adolescents with obesity exhibited notably higher SBP and DBP than those with overweight and normal BMI at both initial and annual assessment. These findings are consistent with previous studies and underscore the importance of arterial hypertension as a key modifiable risk factor for CVD. Furthermore, among cardiovascular risk factors, lipids and lipoproteins hold particular significance. Several studies have demonstrated an association between childhood obesity and elevated concentrations of total cholesterol, LDL, and TG, as well as decreased concentrations of HDL. In our study, subjects with obesity had higher concentrations of LDL, TG, and ApoB, and lower concentrations of HDL and ApoA1 in both assessments compared to subjects with overweight and normal BMI [38]. Insulin resistance is an additional important factor that connects numerous metabolic and cardiovascular complications associated with obesity, while the compensatory hyperinsulinemia marks the initial phases in the development of DM2 [39]. We demonstrated that the concentrations of fasting glucose and insulin, the HOMA-IR index and HbA1C were significantly higher in the participants with obesity than the other two groups. This is a very common finding in intervention programs like the one proposed in our current work and emphasize the well-documented link between obesity and increased risk of insulin resistance and related metabolic and cardiovascular complications.

Therefore, addressing childhood obesity using a lifestyle intervention program of healthy diet, sleep, and exercise is particularly important for the management of comorbidities associated with obesity. 

We have also shown that our lifestyle intervention program effectively reduces obesity rates [5,6]. Following the implementation of this 1-year intervention program, the percentage of participants with obesity decreased significantly by 15.1%, while the percentages of participants with overweight and normal BMI showed a significant increase by 7% and 8%, respectively, due to the transition of children and adolescents with obesity to a lower BMI category. In addition, WHR and body fat percentage significantly decreased in subjects with obesity, while lean mass and muscle mass improved among all individuals. Considering that WHR is an indicator of central obesity and a stronger predictor of cardiovascular risk compared to BMI and WC, the simultaneous reduction in WHR and body fat percentage suggests that the intervention may reduce cardiovascular risk factors in children and adolescents [40]. In line with previous studies, we also observed a significant increase in HDL and decrease in LDL concentrations in all subjects, as well as a reduction in HbA1C, ALT, AST, and γGT concentrations in children with obesity, which highlights the value of a healthier lifestyle for preventing cardiometabolic disorders [5,6].

Interestingly, both TSH and FT4 concentrations showed no significant differences between the three groups at both times of assessment, while T3 concentrations were significantly higher in children with obesity compared to those with overweight and normal BMI. These findings concur partly with previous reports, which demonstrated that elevated TSH concentrations are a frequent finding in children with obesity [41,42]. It has been proposed that the elevation in TSH and T3 concentrations observed in individuals with obesity may be a consequence, rather than a cause, of obesity. The mechanisms behind these changes are unclear, although various theories have been suggested. These include a potential increase in deiodinase activity, as evidenced by the elevated total T3 and FT3 concentrations observed in some individuals. The increased conversion of T4 to T3 in patients with obesity has also been interpreted as a protective mechanism, aiming to counteract fat accumulation by enhancing energy expenditure and basal metabolic rate. This increase correlates positively with total T3 and FT3 concentrations. Leptin, a hormone produced by adipocytes, also influences the activity of deiodinases, promoting the conversion of T4 to T3. Leptin also stimulates TRH and subsequently TSH secretion, thus contributing to mildly elevated TSH concentrations in subjects with obesity. In addition, the resistance to the action of thyroid hormones observed in obesity due to reduced expression of their receptors in adipocytes could explain the compensatory increase in T3 and TSH concentrations [43,44]. 

After 1 year of lifestyle intervention, although obesity rates decreased, there was no significant reduction in serum TSH concentrations, consistent with several other studies. The non-decrease in TSH may also be attributed to the fact that observations were made over a relatively short period. In addition, our study showed a significant decrease in both total T3 and FT4 concentrations at the annual assessment. Similarly, several studies have described a decrease in serum T3 concentrations a few weeks after weight loss, while others reported stable serum T4 concentrations after weight loss. A study by Reinehr et al. in 118 children and adolescents demonstrated a decrease in T3 and FT4 concentrations following a lifestyle intervention program, with TSH concentrations remaining unchanged, as observed in our study too [45,46,47].

Thyroid hormones, including TSH, play a crucial role in affecting cardiometabolic risk factors through multiple mechanisms. T3 and T4 increase basal metabolic rate and thermogenesis by stimulating genes involved in energy expenditure and mitochondrial biogenesis, influencing body weight and fat distribution. T3 enhances lipid metabolism by promoting lipolysis and increasing LDL receptor expression. T3 also improves insulin sensitivity and glucose uptake in peripheral tissues, while T4 has a protective role against insulin resistance. It is also well established that thyroid hormones increase the heart rate and cardiac contractility, enhance the systolic and diastolic heart function, and reduce systemic vascular resistance. Understanding these mechanisms highlights the importance of maintaining thyroid hormone balance to prevent obesity, insulin resistance, and cardiovascular disease in children and adolescents [48,49,50,51].

We also demonstrated that an increase in age by one year leads to a decrease in the concentrations of FT4, TSH, and total T3. This trend of decreasing TSH and thyroid hormone concentrations with age from birth to adulthood (<18 years) is already well documented [52]. In addition, total T3 at baseline was positively associated with Hb1C, glucose, and uric acid concentrations and negatively associated with creatinine, while baseline FT4 was inversely associated with insulin and positively associated with creatinine concentrations and fat mass after controlling for potential confounders, such as age, BMI, puberty, and gender during the 1 year of intervention in all subjects. Thyroid hormones physiologically regulate serum uric acid concentrations by influencing purine nucleotide metabolism and promoting uric acid excretion [53,54]. Furthermore, thyroid hormones affect carbohydrate and lipid metabolism, which can be related to changes in HbA1C, an indicator of long-term glycemic control. Increased T3 concentrations might lead to greater insulin resistance and consequently higher blood glucose concentrations, which are reflected in increased HbA1C [55,56,57]. To the best of our knowledge, the relationship between thyroid hormones, uric acid, and creatinine has not been described in the literature for the pediatric population. Therefore, more prospective studies are required to evaluate this relationship. No associations were found between baseline TSH concentrations and cardiometabolic risk parameters, which do not confirm findings from other studies showing positive associations between TSH and BMI, blood pressure, lipid concentrations, triglycerides, insulin, and HOMA-IR [22,24,25,26]. The differential correlations of FT4 and T3 with insulin sensitivity may seem complex and reflect inconsistencies within the literature regarding the impact of hyperthyroidism on insulin sensitivity and glucose tolerance. The observed differences in our results could be due to variations in study populations, the duration of intervention, and the methods used. In a retrospective study of children aged 2–18 years old, FT4 was negatively associated with triglycerides, while in another study in euthyroid adults, it was negatively correlated with metabolic syndrome parameters [21,22]. In our study, we observed that FT4 was negatively associated with insulin, which was consistent with the results from previous studies in adults [20,58]. The inverse relationship between FT4 and insulin can be explained by the ability of FT4 to enhance glucose metabolism, increase insulin sensitivity, promote lipid metabolism, and reduce inflammation. These factors highlight the beneficial impact of FT4 on metabolic processes, thereby lowering the risk of insulin resistance and related metabolic disorders [51]. These findings also underscore the complex interplay between thyroid hormones and cardiometabolic parameters, highlighting the need for further research studies to understand the mechanisms underlying these relations. Following the 1-year lifestyle intervention, changes in thyroid hormones and TSH concentrations were significantly associated with changes in cardiometabolic factors in all subjects. We observed that changes in TSH concentrations were positively associated with changes in SBP, glucose, cholesterol, and TG and negatively associated with age, while changes in FT4 concentrations were positively correlated with changes in fat mass and creatinine, and negatively correlated with changes in cholesterol, insulin concentrations, fat-free mass (FFM), fat mass (FAM), age, and puberty. A study in 330 euthyroid children with overweight and obesity demonstrated that changes in serum TSH concentrations (but not FT4) were significantly associated with changes in cardiovascular risk parameters in children with successful weight loss. It is worth noting that these alterations were not a result of weight loss, given that changes in the BMI z-score were not associated with changes in cardiovascular risk parameters [32]. In another study by Aeberli et al., changes in TSH concentrations during an intensive, well-controlled inpatient intervention were also associated with changes in cardiometabolic risk factors independent of changes in body weight or composition in children and adolescents with obesity [29]. Furthermore, in all subjects, changes in T3 concentrations were positively correlated with changes in glucose, uric acid, TG, and HbA1C concentrations and negatively associated with changes in creatinine concentrations. These findings align with the study by Liu et al., who described that changes in FT3 and total T3 concentrations were positively correlated with changes in cardiometabolic parameters, including blood pressure, glucose, insulin, and TG [59].

Strengths of our study include the significant number of participants, the high percentage of participants with obesity, the investigation of multiple laboratory and clinical parameters, and the detailed description of the sample characteristics, all of which enhance the statistical power and reliability of the study results. However, a limitation of our study is that the 1-year duration of the intervention program may be considered relatively short. Another limitation of this prospective study is the lack of a randomized control group without intervention, which is owing to the fact that all patients were consecutive attendees at our Center for the Prevention and Management of Childhood Obesity. Another limitation of our study is the inclusion of participants at various stages of sexual development (pre-, peri-, mid-pubertal, and mature adolescents), which can introduce variability in metabolic data. The hormonal and metabolic changes associated with puberty may influence the interpretation of our findings. While we controlled for pubertal status in our statistical analysis to mitigate this issue, we acknowledge that this does not entirely eliminate the potential for a confounding effect. This variability in pubertal stages is an important consideration and should be taken into account when interpreting our results. In addition, we recognize that the observed differences and changes in thyroid function within our study are relatively minor and may have limited clinical significance. These alterations do not mirror the pronounced variations typically seen in conditions, such as Graves’ disease or primary hypothyroidism, which have been the focus of most previous research on insulin production and action. This distinction is important for interpreting our results and highlights the need for cautious application of our findings in clinical practice.

Self-reported data on lifestyle changes, such as diet, sleep, and physical activity, may be subject to recall bias. Participants might overreport positive behaviors or underreport negative ones, which could affect the accuracy of the data collected. To mitigate this, we utilized trained personnel to conduct interviews. Despite these limitations, we believe our study contributes important preliminary data that underscore the need for targeted interventions in managing childhood obesity and associated cardiometabolic risks. To address the above issues, future research should focus on longitudinal studies to determine the long-term impact of thyroid hormone changes on cardiometabolic parameters. In addition, exploring genetic factors that influence these relationships could provide insights into individual risk profiles. Finally, extending the intervention time will make the findings more relevant.

## 5. Conclusions

In summary, in children and adolescents with overweight and obesity, thyroid hormones are associated with indices conferring cardiometabolic risk. Specifically, changes in FT4, T3, and TSH concentrations are also associated with changes in key cardiometabolic parameters, such as cholesterol, glucose, and triglyceride concentrations. Our findings further support previous studies suggesting that thyroid hormones act as mediators in lipid regulation and glucose metabolism. Although a definitive cause-and-effect relationship cannot be established, our results highlight the potential clinical relevance of monitoring thyroid function as part of a comprehensive strategy for managing obesity and associated cardiometabolic risk in this population.

## Figures and Tables

**Table 1:** Clinical characteristics and anthropometric (A), hematologic (B), biochemical (C), and endocrinologic parameters (D) in all subjects at initial and annual assessment.

	Initial Assessment				Annual Assessment				
	Obesity N (%) 727 (55.45%)	Overweight N (%) 384 (29.29%)	Normal BMI N (%) 200 (15.26%)	*p*-Value	Obesity N (%) 529 (40.35%)	Overweight N (%) 476 (36.31%)	Normal BMI N (%) 306 (23.34%)	*p*-Value	*p*_between time points_
**A. Anthropometric parameters**									
Age, years	10.20 ± 3.03	10.16 ± 2.65	9.62 ± 2.95	0.01	11.23 ± 3.11	11.09 ± 2.74	11.00 ± 2.82	NS	0.01/0.01/0.01
Pubertal status	405 (55.71%)	218 (56.77%)	109 (54.5%)	NS	147 (27.79%)	131 (27.52%)	84 (27.45%)	NS	0.01/0.01/0.01
Prepubertal Pubertal	313 (43.05%)	166 (43.23%)	85 (42.5%)		236(44.61%)	212 (44.54%)	154(50.33%)		
Height, cm	145.04 ± 18.22	143.39 ± 14.46	137.23 ± 17.72	0.01	151.28 ± 17.04	149.13 ± 15.26	145.64 ± 15.97	0.01	0.01/0.01/0.01
BW, kg	62.09 ± 22.07	49.29 ± 13.80	37.21 ± 13.34	0.01	67.31 ± 21.66	53.73 ± 15.02	44.07 ± 14.10	0.01	0.01/0.01/0.01
BMI, kg/m^2^	28.28 ± 4.45	23.39 ± 2.24	18.99 ± 2.77	0.01	28.47 ± 4.42	23.57 ± 2.41	20.14 ± 3.05	0.01	0.01/0.01/NS
WC, cm	87.38 ± 13.56	76.73 ± 9.07	65.86 ± 10.55	0.01	90.44 ± 14.26	80.01 ± 9.15	69.96 ± 9.61	0.01	NS/NS/0.01
HC, cm	93.06 ± 15.21	83.98 ± 10.88	74.35 ± 12.20	0.01	97.46 ± 14.69	88.00 ± 10.27	80.70 ± 11.30	0.01	0.01/0.01/0.01
WHR	0.95 ± 0.11	0.92 ± 0.11	0.89 ± 0.11	0.01	0.93 ± 0.07	0.91 ± 0.07	0.87 ± 0.07	0.01	0.05/NS/0.05
**B. Hematologic parameters**									
WBC, ×10^9^/L	7.66 ± 1.96	7.31 ± 1.87	7.11 ± 2.05	0.01	7.65 ± 1.89	7.06 ± 1.74	6.81 ± 1.85	0.01	NS/0.01/NS
RBC, ×10^12^/L	5.04 ± 0.44	4.95 ± 0.40	4.94 ± 0.45	0.01	5.06 ± 0.45	4.99 ± 0.44	4.94 ± 0.42	0.01	NS/NS/NS
HGB, g/dL	12.93 ± 0.96	12.91 ± 0.87	12.83 ± 0.97	NS	12.95 ± 1.03	13.03 ± 0.98	12.92 ± 0.98	NS	0.01/NS/NS
HCT, %	40.431 ± 2.74	40.24 ± 2.36	40.03 ± 2.87	NS	40.54 ± 2.99	40.59 ± 2.80	40.25 ± 2.80	NS	NS/NS/NS
PLT, ×10^9^/L	307.82 ± 66.44	302.33 ± 65.90	298.08 ± 66.75	NS	299.15 ± 63.93	285.05 ± 60.91	290.19 ± 68.14	0.05	0.01/0.01/0.01
ESR, mm/h	19.45 ± 12.75	18.10 ± 11.97	15.16 ± 10.47	0.01	18.36 ± 12.57	16.30 ± 11.02	14.11 ± 9.89	0.01	0.01/NS/0.05
**C. Biochemical parameters**									
Ferritin, μg/L	57.99 ± 33.34	53.10 ± 40.04	46.18 ± 28.21	0.01	53.42 ± 32.71	52.03 ± 41.82	45.02 ± 25.82	0.01	0.01/0.01/0.01
Folic acid, ng/mL	11.62 ± 4.49	11.24 ± 4.56	11.13 ± 4.33	NS	8.94 ± 4.24	9.62 ± 4.56	9.39 ± 5.29	NS	0.01/0.01/0.01
Urea, mg/dL	28.81 ± 6.47	28.52 ± 6.13	29.36 ± 6.54	NS	28.55 ± 6.38	28.13 ± 6.01	27.36 ± 6.32	0.05	NS/0.05/NS
Creatinine, mg/dL	0.50 ± 0.12	0.49 ± 0.12	0.47 ± 0.11	NS	0.54 ± 0.13	0.53 ± 0.12	0.52 ± 0.12	NS	0.01/0.01/0.01
AST, U/L	23.84 ± 6.58	23.57 ± 6.86	24.34 ± 5.62	NS	22.52 ± 6.67	22.11 ± 6.49	22.78 ± 7.02	NS	0.01/0.01/0.01
ALT, U/L	22.83 ± 13.27	18.99 ± 9.66	16.12 ± 5.50	0.01	22.17 ± 13.84	17.76 ± 7.74	16.86 ± 6.94	0.01	0.01/0.01/NS
γGT, U/L	15.35 ± 7.06	12.73 ± 4.51	10.97 ± 3.27	0.01	14.85 ± 5.80	12.53 ± 4.72	11.39 ± 7.15	0.01	0.01/0.01/NS
ALP, U/L	235.80 ± 77.64	223.69 ± 76.58	229.65 ± 71.72	NS	220.83 ± 77.59	218.28 ± 80.78	218.13 ± 83.28	NS	0.01/NS/NS
Phosphorus, mg/dL	4.74 ± 0.54	4.68 ± 0.57	4.78 ± 0.50	NS	4.68 ± 0.55	4.68 ± 0.54	4.67 ± 0.54	NS	0.01/NS/0.01
Albumin, g/dL	4.63 ± 0.21	4.64 ± 0.22	4.63 ± 0.23	NS	4.59 ± 0.22	4.63 ± 0.21	4.62 ± 0.22	0.01	NS/NS/NS
Uric acid, mg/dL	4.94 ± 1.12	4.43 ± 0.88	3.97 ± 0.84	0.01	4.98 ± 1.17	4.54 ± 0.99	4.12 ± 0.90	0.01	NS/NS/NS
Potassium, mmol/L	4.42 ± 0.30	4.39 ± 0.31	4.41 ± 0.35	NS	4.42 ± 0.29	4.41 ± 0.33	4.39 ± 0.27	NS	NS/NS/NS/
Sodium, mmol/L	140.41 ± 1.57	140.26 ± 1.55	140.2 ± 1.57	NS	140.28 ± 1.53	140.25 ± 1.56	140.35 ± 1.55	NS	NS/NS/NS
Calcium, mmol/L	9.9 ± 0.35	9.85 ± 0.37	9.82 ± 0.38	0.01	9.77 ± 0.35	9.78 ± 0.34	9.77 ± 0.34	NS	0.01/0.01/0.01
**D. Endocrinologic parameters**									
FT4, ng/dL	1.11 ± 0.14	1.12 ± 0.14	1.13 ± 0.15	NS	1.09 ± 0.14	1.09 ± 0.15	1.09 ± 0.15	NS	0.05/0.01/0.05
T3, ng/dL	148.37 ± 32.12	138.90 ± 25.37	135.62 ± 27.40	0.01	142.20 ± 29.65	135.19 ± 28.44	133.47 ± 27.11	0.01	0.01/0.01/0.05
TSH, μUI/mL	2.98 ± 1.44	2.865 ± 1.38	2.94 ± 1.51	NS	2.98 ± 1.49	2.85 ± 1.47	2.80 ± 1.50	NS	NS/NS/NS
IGF-I, ng/mL	286.04 ± 167.59	302.07 ± 170.72	286.75 ± 182.33	NS	343.78 ± 193.97	341.62 ± 191.6	329.33 ± 171.97	NS	0.01/0.01/0.01
IGFBP3, μg/mL	5.08 ± 1.05	4.95 ± 0.96	4.76 ± 1.06	0.01	5.32 ± 1.09	5.19 ± 1.08	5.05 ± 1.11	0.05	0.01/0.01/0.01
Prolactin, ng/mL	11.79 ± 6.93	12.31 ± 7.02	12.74 ± 7.88	NS	12.51 ± 8.32	13.20 ± 9.66	13.53 ± 9.01	NS	0.05/0.05/0.01
LH, IU/L	1.96 ± 3.99	2.72 ± 7.92	1.69 ± 2.87	NS	2.68 ± 3.55	2.78 ± 4.89	2.92 ± 3.45	NS	0.01/0.01/0.01
FSH, IU/L	2.41 ± 2.09	2.61 ± 2.23	2.77 ± 2.21	0.05	2.82 ± 2.08	3.08 ± 2.26	3.6 ± 2.44	0.01	0.01/0.01/0.01
PTH, pg/mL	34.98 ± 12.62	33.11 ± 13.29	35.12 ± 13.65	0.05	36.61 ± 12.67	35.49 ± 12.00	35.16 ± 12.27	NS	0.05/0.05/NS
Total 25-OH-vitamin D, ng/mL	22.69 ± 9.93	24.17 ± 9.55	25.86 ± 10.58	0.01	24.05 ± 8.75	26.28 ± 9.40	27.77 ± 10.35	0.01	0.01/0.01/0.01
ACTH, pg/mL	31.77 ± 25.56	27.33 ± 21.56	24.28 ± 15.86	0.01	30.50 ± 22.85	29.23 ± 21.79	28.4 ± 23.07	NS	NS/0.01/0.01
Cortisol, μg/dL	14.26 ± 6.23	14.43 ± 6.56	13.88 ± 6.09	NS	13.10 ± 5.72	13.22 ± 5.70	13.37 ± 5.33	NS	0.01/NS/NS

Abbreviations: ALP, alkaline phosphatase; ALT, alanine transaminase; ApoA1, apolipoprotein A1; AST, aspartate aminotransferase; BW, body weight; DBP, diastolic blood pressure; ESR, erythrocyte sedimentation rate; FSH, follicle-stimulating hormone; FT_4_, free thyroxine; γ-GT, serum γ-glutamyl-tranferase; HC, hip circumference; HCT, hematocrit; HDL, high-density lipoprotein; HGB, hemoglobin; IGF-I, insulin-like growth factor I; IGFBP3, IGF-binding protein 3; LDL, low-density lipoprotein; LH, luteinizing hormone; PLT, platelet count; PTH, parathormone; RBC, red blood cell count; T_3_, triiodothyronine; total 25-OH-Vitamin D, total 25-hydroxyvitamin D; TSH, thyroid-stimulating hormone; WBC, white blood cell count; WHR, waist-to-hip ratio. Continuous variables are reported as means ± standard deviation (SD), while categorical variables are shown as frequencies (percentages). To compare the three BMI categories, ANOVA was used for normally distributed variables, Kruskal–Wallis H test for skewed variables, and Pearson’s chi-square test for categorical data. Changes between the two time points were assessed with the Paired T-test for normally distributed variables, the Wilcoxon signed-rank test for skewed variables, and McNemar’s Bowker test for categorical variables. Statistical significance was set at *p* < 0.05, as shown in the table, with results rounded to 0.05. Stronger significance is indicated by *p* < 0.01, rounded to 0.01 in the table. Non-significant differences are labeled as NS (*p* > 0.05).

**Table 2:** Cardiometabolic risk factors (A) and body composition parameters (B) in all subjects at initial and annual assessment.

	Initial Assessment				Annual Assessment				
	Obesity N (%) 727 (55.45%)	Overweight N (%) 384 (29.29%)	Normal BMI N (%) 200 (15.26%)	*p*-Value	Obesity N (%) 529 (40.35%)	Overweight N (%) 476 (36.31%)	Normal BMI N (%) 306 (23.34%)	*p*-Value	*p*_between time points_
**A. Cardiometabolic risk factors**									
Cholesterol, mg/dL	157.96 ± 28.52	158.12 ± 27.35	158.54 ± 26.57	NS	157.89 ± 28.09	155.95 ± 25.71	155.51 ± 24.65	NS	NS/NS/0.05
TG, mg/dL	83.69 ± 51.01	74.28 ± 57.70	61.19 ± 28.82	0.01	88.17 ± 54.41	73.25 ± 42.97	64.76 ± 29.47	0.01	NS/NS/NS
HDL mg/dL	49.26 ± 10.99	53.94 ± 12.68	59.73 ± 13.34	0.01	51.42 ± 12.23	55.94 ± 13.25	59.83 ± 12.91	0.01	0.01/0.01/0.05
LDL mg/dL	92.11 ± 25.30	90.25 ± 23.72	87.15 ± 22.86	0.05	89.63 ± 24.75	85.88 ± 22.54	83.43 ± 20.92	0.01	0.01/0.01/0.01
ApoA1, mg/dL	138.60 ± 20.24	141.88 ± 19.23	149.06 ± 21.12	0.01	139.26 ± 22.21	141.65 ± 21.0	145.44 ± 20.64	0.01	NS/NS/NS
ApoB, mg/dL	76.27 ± 18.30	73.48 ± 16.55	71.77 ± 14.76	0.01	75.54 ± 18.11	73.27 ± 15.60	71.23 ± 14.86	0.05	NS/NS/NS
Lp(a), mg/dL	16.59 ± 24.74	15.44 ± 21.21	16.64 ± 22.21	NS	16.80 ± 25.69	18.65 ± 25.40	16.37 ± 23.16	NS	0.05/0.05/0.05
Glucose, mg/dL	81.33 ± 10.07	79.25 ± 8.53	79.02 ± 7.62	0.01	82.26 ± 7.24	82 ± 7.65	80.49 ± 7.29	0.05	0.01/0.01/0.01
Insulin, μUI/mL	19.29 ± 37.52	12.49 ± 7.12	9.06 ± 5.29	0.01	18.78 ± 10.84	13.53 ± 6.96	11.15 ± 6.81	0.01	NS/0.05/0.01
HbA_1c_, %	5.26 ± 0.25	5.21 ± 0.24	5.15 ± 0.23	0.01	5.25 ± 0.23	5.18 ± 0.25	5.16 ± 0.23	0.01	0.01/NS/NS
HOMA-IR	4.35 ± 18.34	2.46 ± 1.47	1.82 ± 1.12	0.01	3.86 ± 2.37	2.77 ± 1.52	2.24 ± 1.41	0.01	NS/0.01/0.01
DBP, mmHg	67.54 ± 10.99	64.66 ± 9.14	63.18 ± 9.29	0.01	68.38 ± 9.45	66.19 ± 9.16	63.83 ± 8.18	0.01	NS/NS/NS
SBP, mmHg	113.54 ± 12.83	108.75 ± 10.79	105.07 ± 10.34	0.01	114.26 ± 11.97	109.519 ± 10.72	105.61 ± 9.94	0.01	NS/0.05/NS
**B. Body Composition**									
Fat (%)	37.38 ± 6.13	31.63 ± 4.59	25.83 ± 5.55	0.01	37.05 ± 6.64	31.40 ± 4.90	27.02 ± 5.71	0.01	0.01/0.01/NS
Fat Mass (kg)	23.97 ± 10.52	15.77 ± 5.65	10.30 ± 5.18	0.01	25.80 ± 10.78	17.18 ± 5.84	12.27 ± 5.56	0.01	0.01/0.01/0.01
Muscle Mass (PMM) (kg)	36.74 ± 11.75	31.76 ± 8.80	26.79 ± 8.98	0.01	40.34 ± 12.25	35.27 ± 10.09	30.44 ± 9.20	0.01	0.01/0.01/0.01
Bone Mass (kg)	2.00 ± 0.59	1.75 ± 0.45	1.49 ± 0.45	0.01	2.18 ± 0.61	1.92 ± 0.51	1.67 ± 0.46	0.01	0.01/0.01/0.01
Fat Free Mass (FFM) (kg)	38.74 ± 12.34	33.53 ± 9.22	28.29 ± 9.44	0.01	42.54 ± 12.87	37.2 ± 10.60	32.11 ± 9.66	0.01	0.01/0.01/0.01
Total Body Water (TBW) (%)	28.35 ± 9.01	24.53 ± 6.76	20.70 ± 6.88	0.01	31.13 ± 9.38	27.23 ± 7.76	23.51 ± 7.07	0.01	0.01/0.01/0.01
Basal Metabolic Rate (BMR) (Kilojoule)	6525.38 ± 1333.28	5764.33 ± 913.56	5109.23 ± 812.15	0.01	6885.51 ± 1378.22	6167.64 ± 1091.76	5462.03 ± 903.47	0.01	0.01/0.01/0.01

Abbreviations: ApoA1, apolipoprotein A1; ApoB, apolipoprotein B; HDL, high-density lipoprotein; HOMA-IR, homeostatic model assessment for insulin resistance; LDL, low-density lipoprotein; Lp(a), Lipoprotein (a); TG, triglycerides. Variables are expressed as means ± standard deviation (SD); *p*-values were determined by using the ANOVA test for normally distributed variables and the Kruskal–Wallis H test for skewed variables when comparing the three BMI categories; *p* values between time points were derived by comparisons between the two assessments using paired T test or Wilcoxon’s signed rank test for skewed data. Statistical significance was set at *p* < 0.05, as shown in the table, with results rounded to 0.05. Stronger significance is indicated by *p* < 0.01, rounded to 0.01 in the table. Non-significant differences are labeled as NS (*p* > 0.05).

**Table 3:** Mixed regression analysis of thyreotropin and thyroid hormones compared with antropometric parameters, body composition parameters, biochemical parameters, and cardiometabolic risk factors after controlling for potential confounders, such as age, BMI, puberty, and gender.

	FT4 (β, 95% CI)	TSH (β, 95% CI)	T3 (β, 95% CI)
FATM, kg	0.003 (0.002, 0.005)	0.013 (−0.0061, 0.0322)	−0.312 (−0.657, 0.033)
PMM, kg	0.122 (−0.087, 0.331)	−1.409 (−3.6819, 0.8645)	40.411 (−0.062, 80.884)
FFM, kg	−0.115 (−0.314, 0.084)	1.362 (−0.8018, 3.5251)	−38.485 (−77.015, 0.046)
BMR, kJ	−0.0001 (−0.0001, 0.0001)	−0.0003 (−0.0009, 0.0004)	−0.001 (−0.013, 0.010)
Glucose, mg/dL	−0.0004 (−0.001, 0.0005)	0.008 (−0.0027, 0.0178)	0.338 (0.153, 0.522)
Cholesterol, mg/dL	−0.0007 (−0.002, 0.0005)	0.009 (−0.0045, 0.0224)	0.069 (−0.176, 0.314)
Insulin, μUI/mL	−0.0005 (−0.001, −0.0002)	0.001 (−0.0024, 0.0043)	0.022 (−0.042, 0.085)
Uric acid, mg/dL	0.008 (−0.0002, 0.017)	−0.041 (−0.132, 0.050)	4.293 (2.662, 5.924)
HDL, mg/dL	0.0007 (−0.001, 0.002)	−0.007 (−0.022, 0.008)	−0.226 (−0.498, 0.047)
LDL, mg/dL	0.0004 (−0.001, 0.002)	−0.005 (−0.019, 0.008)	−0.063 (−0.308, 0.182)
TG, mg/dL	−0.0001 (−0.0003, 0.0002)	0.002 (−0.0004, 0.005)	0.021 (−0.030, 0.071)
HbA1C, %	0.017 (−0.013, 0.05)	0.112 (−0.222, 0.445)	9.107 (3.104, 15.109)
SBP, mmHg	0.0001 (−0.001, 0.001)	0.008 (−0.001, 0.016)	0.026 (−0.133, 0.185)
DBP, mmHg	−0.0003 (−0.001, 0.0004)	0.001 (−0.008, 0.011)	0.047 (−0.121, 0.215)
Creatinine, mg/dL	0.171 (0.08, 0.262)	0.639 (−0.350, 1.627)	−64.537 (−82.242, −46.833)
Hip, cm	0.0003 (−0.001, 0.002)	−0.001 (−0.015, 0.012)	0.184 (−0.058, 0.427)
Waist, cm	−0.001 (−0.002, 0.0004)	−0.003 (−0.017, 0.01)	0.153 (−0.087, 0.393)
Pubertal Status (Puberty)	−0.043 (−0.064, −0.022)	0.132 (−0.096, 0.359)	4.258 (0.210, 8.306)
Time	−0.009 (−0.022, 0.004)	−0.023 (−0.164, 0.118)	−0.783 (−3.217, 1.650)
Age, years	−0.009 (−0.015, −0.002)	−0.079 (−0.152, −0.005)	−3.255 (−4.571, −1.939)
BMI Obesity, kg/m^2^	−0.005 (−0.034, 0.023)	0.036 (−0.271, 0.342)	3.754 (−1.687, 9.195)
BMI Overweight, kg/m^2^	−0.007 (−0.029, 0.016)	−0.135 (−0.381, 0.111)	−1.724 (−6.045, 2.598)
Gender (Female)	−0.058 (−0.099, −0.018)	−0.145 (−0.582, 0.292)	−2.090 (−9.914, 5.734)

Abbreviations: BMI, body mass index; BMR, basal metabolic rate; DBP, diastolic blood pressure; FT4, free thyroxine; HbA1C, hemoglobin A1C; HDL, high density lipoprotein; Hip, hip circumference; LDL, low density lipoprotein; PMM, muscle mass; SBP, systolic blood pressure; T3, triiodothyronine; TG, triglyceride; TSH, thyroid-stimulating hormone; Waist, waist circumference; statistical significance was set at *p* < 0.05.

**Table 4:** Stepwise regression analysis among changes in FT4 (A), TSH (B), T3 (C), and cardiometabolic parameters for all subjects after controlling for potential confounders, such as age, BMI, puberty, and gender during the 1 year of intervention.

	Adj. Beta	95% CI	*p*-Value
**A. FT4**			
FATM, kg	0.003	0.001, 0.004	0.01
BMR, kJ	−0.00003	−0.0001, −0.00002	0.01
Cholesterol, mg/dL	−0.0004	−0.001, −0.0001	0.05
Insulin, μUI/mL	−0.001	−0.001, −0.0003	0.01
Creatinine, mg/dL	0.198	0.110, 0.284	0.01
Pubertal Status (Pubertal)	−0.044	−0.065, −0.024	0.01
Age (Years)	−0.009	−0.014, −0.004	0.01
Gender (Female)	−0.043	−0.064, −0.022	0.01
BMI Obese, kg/m^2^	−0.009	−0.035, 0.017	NS
BMI Overweight, kg/m^2^	−0.0104	−0.032, 0.011	NS
**B. TSH**			
Glucose, mg/dL	0.010	0.001, 0.019	0.05
Cholesterol, mg/dL	0.004	0.001, 0.007	0.05
TG, mg/dL	0.003	0.001, 0.005	0.01
SBP, mm Hg	0.008	0.001, 0.016	0.05
Age (Years)	−0.056	−0.088, −0.023	0.01
Gender (Female)	0.016	−0.162, 0.194	NS
BMI Obese, kg/m^2^	0.004	−0.229, 0.237	NS
BMI Overweight, kg/m^2^	−0.154	−0.379, 0.072	NS
**C. T3**			
PMM, Kg	43.372	3.292, 83.452	0.05
FFM, Kg	−41.384	−79.589, −3.180	0.05
Glucose, mg/dL	0.370	0.204, 0.536	0.01
Uric Acid, mg/dL	4.463	2.861, 6.066	0.01
HDL, mg/dL	−0.178	−0.306, −0.050	0.01
TG, mg/dL	0.033	0.003, 0.063	0.05
HbA1c, (%)	9.551	3.614, 15.487	0.01
Creatinine, mg/dL	−64.833	−82.059, −47.607	0.01
Puberty Status (Pubertal)	4.317	0.314, 8.319	0.05
Age (Years)	−2.975	−4.015, −1.935	0.01
Gender (Female)	−1.718	−5.670, 2.234	NS
BMI Obese, kg/m^2^	5.537	0.906, 10.168	0.05
BMI Overweight, kg/m^2^	−0.530	−4.633, 3.574	NS

Abbreviations: BMI, body mass index; BMR, basal metabolic rate; DBP, diastolic blood pressure; FATM, fat mass; FFM, free fat mass; FT4, free thyroxine; HbA1C, hemoglobin A1C; HDL, high density lipoprotein; LDL, low density lipoprotein; PMM, muscle mass; SBP, systolic blood pressure; T3, triiodothyronine; TG, triglyceride; TSH, thyroid-stimulating hormone. Statistical significance was set at *p* < 0.05, as shown in the table, with results rounded to 0.05. Stronger significance is indicated by *p* < 0.01, rounded to 0.01 in the table. Non-significant differences are labeled as NS (*p* > 0.05).

**Table 5:** Associations among changes in FT4 (A), TSH (B), T3 (C), and cardiometabolic parameters in subjects with obesity after controlling for potential confounders, such as age, puberty, and gender during the 1 year of intervention.

	Adj. Beta	95%CI	*p*-Value
**A. T4**			
FATM, kg	0.002	0.0004, 0.003	0.05
ffm, kg	−0.003	−0.004, −0.0008	0.01
Cholesterol, mg/dL	−0.0004	−0.0008, −0.00002	0.05
Insulin, μUI/mL	−0.0006	−0.0008, −0.0003	0.01
Creatinine, mg/dL	0.236	0.118, 0.354	0.01
Pubertal Status (Puberty)	−0.032	−0.062, −0.002	0.05
Age, years	−0.012	−0.019, −0.004	0.01
Gender (Female)	−0.027	−0.049, −0.004	0.05
**B. TSH**			
TG, mg/dL	0.004	0.002, 0.006	0.01
SBP, mmHg	0.012	0.001, 0.022	0.05
Pubertal Status (Puberty)	0.271	−0.059, 0.601	NS
Age, years	−0.114	−0.175, −0.052	0.01
Gender (Female)	−0.261	−0.519, −0.003	NS
**C. T3**			
Glucose, mg/dL	0.410	0.166, 0.654	0.01
Uric acid, mg/dL	5.647	3.411, 7.883	0.01
TG, mg/dL	0.051	0.010, 0.092	0.05
Creatinine, mg/dL	−66.117	−92.619, −39.616	0.01
Pubertal Status (Puberty)	4.462	−2.098, 11.022	NS
Age, years	−3.330	−4.676, −1.984	0.01
Gender (Female)	1.474	−3.689, 6.637	NS

Abbreviations: BMI, body mass index; FATM, fat mass; FFM, free fat mass; FT4, free thyroxine; SBP, systolic blood pressure; T3, triiodothyronine; TG, triglyceride; TSH, thyroid-stimulating hormone; Statistical significance was set at *p* < 0.05, as shown in the table, with results rounded to 0.05. Stronger significance is indicated by *p* < 0.01, rounded to 0.01 in the table. Non-significant differences are labeled as NS (*p* > 0.05).

**Table 6:** Associations among changes in FT4 (A), TSH (B), T3 (C) and cardiometabolic parameters in subjects with Overweight after controlling for potential confounders, such as age, pubertal and gender during the 1 year of intervention.

	Adj. Beta	95%CI	*p*-Value
**A. T4**			
FATM, kg	0.005	0.0006, 0.008	0.05
BMR, kJ	−0.00004	−0.00007, −0.00001	0.01
Glucose, mg/dL	−0.002	−0.003, −0.0002	0.05
TG, mg/dL	−0.0003	−0.0006, −0.00003	0.05
Creatinine, mg/dL	0.179	0.021, 0.337	0.05
Puberty	−0.035	−0.071, 0.0006	NS
Age, years	−0.009	−0.018, 0.0007	NS
Gender (Female)	−0.054	−0.095, −0.014	0.01
**B. TSH**			
Cholesterol, mg/dL	0.005	0.0002, 0.009	0.05
Time	−0.214	−0.422, −0.007	0.05
Age, years	0.005	−0.058, 0.067	NS
Gender (Female)	0.215	−0.067, 0.497	NS
Puberty	0.064	−0.254, 0.381	NS
**C. T3**			
FATM, kg	−1.181	−1.783, −0.578	0.01
Glucose, mg/dL	0.331	0.071, 0.592	0.05
Uricacid, mg/dL	4.221	1.658, 6.784	0.01
HDL, mg/dL	−0.224	−0.400, −0.048	0.05
HbA1C, %	11.922	2.954, 20.889	0.01
Creatinine, mg/dL	−62.774	−87.838, −37.709	0.01
Hip, cm	0.385	0.027, 0.743	0.05
Age, years	−2.242	−3.839, −0.644	0.01
Puberty	2.903	−2.576, 8.382	NS
Gender (Female)	1.114	−3.927, 6.155	NS

Abbreviations: BMI, body mass index; BMR, basal metabolic rate; FATM, fat mass; FT4, free thyroxine; HbA1C, hemoglobin A1C; HDL, high density lipoprotein; Hip, hip circumference; T3, triiodothyronine; TG, triglyceride; TSH, thyroid-stimulating hormone; Statistical significance at *p* < 0.05 (rounded to 0.05 in the Table), while strong significance of *p* < 0.01 (rounded to 0.01 in the Table) is also noted. NS: non-significant (*p* > 0.05) difference statistical significance was set at *p* < 0.05.

**Table 7:** Associations among changes in FT4 (A), TSH (B), T3 (C), and cardiometabolic parameters in subjects with normal BMI after controlling for potential confounders, such as age, pubertal status, and gender during the 1 year of intervention.

	Adj. Beta	95%CI	*p*-Value
**A. T4**			
Insulin, μUI/mL	−0.004	−0.007, −0.001	0.01
Puberty	−0.108	−0.156, −0.059	0.01
Age, years	0.004	−0.005, 0.013	NS
Gender (Female)	0.017	−0.027, 0.060	NS
**B. TSH**			
Cholesterol, mg/dL	0.008	0.0001, 0.015	<0.05
Insulin, μUI/mL	0.051	0.015, 0.087	<0.01
TG, mg/dL	0.007	0.0003, 0.0135	<0.05
Age, years	−0.086	−0.181, 0.008	NS
Gender (Female)	−0.034	−0.492, 0.424	NS
Puberty	−0.147	−0.661, 0.366	NS
**C. T3**			
Insulin, μUI/mL	0.930	0.403, 1.457	<0.01
HDL, mg/dL	−0.271	−0.498, −0.043	<0.05
HbA1c, %	14.050	0.983, 27.117	<0.05
SBP, mmHg	0.367	0.031, 0.704	<0.05
Creatinine, mg/dL	−69.670	−106.004, −33.337	<0.01
Age, years	−3.189	−5.018, −1.362	<0.01
Gender (Female)	−4.891	−12.418, 2.635	NS
Puberty	4.124	−4.178, 12.427	NS

Abbreviations: FT4, free thyroxine; HbA1C, hemoglobin A1C; HDL, SBP, systolic blood pressure; T3, triiodothyronine; TG, triglyceride; TSH, thyroid-stimulating hormone; Statistical significance was set at *p* < 0.05, as shown in the table, with results rounded to 0.05. Stronger significance is indicated by *p* < 0.01, rounded to 0.01 in the table. Non-significant differences are labeled as NS (*p* > 0.05).

## Data Availability

The data from this study can be obtained by contacting the corresponding author. They are not available for public access due to privacy concerns.

## Supplementary Materials

The following supporting information can be downloaded at https://www.mdpi.com/article/10.3390/nu16162650/s1: Table S1: Cardiometabolic risk factors, body composition parameters and thyroid hormones at initial and annual assessment: comparisons according to gender (irrespective of pubertal status); Table S2: Cardiometabolic risk factors and thyroid hormones at initial and annual assessment: comparisons according to pubertal status (irrespective of gender).
