# Supplementary material for: The Impact of Thyroid Hormones on Cardiometabolic Risk in Children and Adolescents with Obesity, Overweight and Normal Body Mass Index (BMI): A One-Year Intervention Study
Source: Nutrients. 2024 Aug 11;16(16):2650. doi: 10.3390/nu16162650 (PMC11357135; doi:10.3390/nu16162650)
Supplement: Supplementary file 1 [file nutrients-16-02650-s001.zip › nutrients-3068648-supplementary.pdf]

Table S1: Cardiometabolic risk factors, body composition parameters and thyroid hormones at initial and annual assessment: Comparisons according to gender (irrespective of pubertal status)

	Initial Assessment			Annual Assessment			
	**Boys**	**Girls**	**P within**	**Boys**	**Girls**	**P within**	**P between timepoints (boys vs girls)**
SBP, mmHg	111.70±12.67	109.99±11.91	**<0.05**	112.65±12.41	108.76±10.55	**<0.05**	NS/NS
DBP, mmHg	65.99±10.62	66.03±10.12	NS	66.73±9.42	66.32±9.06	NS	NS/NS
Cholesterol, mg/dL	160.28±28.54	156.10±27.11	**<0.05**	157.83±26.86	155.49±25.99	NS	**<0.05**/NS
HDL, mg/dL	52.97±13.36	51.52±11.54	NS	55.26±13.94	54.93±12.48	NS	**<0.05/<0.05**
LDL, mg/dL	92.15±25.04	89.59±24.02	**<0.05**	87.34±23.29	86.25±23.09	NS	**<0.05/<0.05**
Lp(a), mg/dL	16.86±23.93	15.72±22.86	NS	17.75±25.56	17.05±24.49	NS	**<0.05/<0.05**
TG, mg/dL	77.17±52.89	77.84±49.29	NS	78.76±51.20	75.55±41.22	NS	NS/NS
ApoB, mg/dL	75.53±17.88	74.08±16.88	NS	74.17±16.58	73.20±16.48	NS	**<0.05**/NS
Apo A1, mg/dL	143.12±20.97	139.39±19.73	**<0.05**	143.23±22.38	140.19±20.62	**<0.05**	NS/NS
Glucose, mg/dL	81.57±9.94	79.29±8.65	**<0.05**	83.03±7.35	80.59±7.33	**<0.05**	**<0.05/<0.05**
Insulin, μUI/mL	16.04±39.79	15.48±11.39	NS	14.48±8,31	15.49±9.91	NS	NS/**<0.05**
HbA1C %	5.24±0.25	5.21±0.24	**<0.05**	5.21±0.24	5.20±0.23	NS	**<0.05/<0.05**
Homa- IR	3.74±19.78	3.12±2.56	NS	3.00±1.82	3.13±2.12	NS	NS/<**0.05**
TSH,μU/mL	2.99±1.48	2.89±1.38	NS	2.89±1.48	2.88±1.49	NS	**<0.05**/NS

FT4, ng/dL	1.12±0.14	1.11±0.15	NS	1.09±0.15	1.09±0.14	NS	**<0.05/<0.05**
T3, ng/dL	142.79±25.16	144.48±33.91	NS	136.78±25.61	138.17±31.47	NS	**<0.05/<0.05**
Fat Percentage (FATP), %	33.40±7.79	34.38±6.29	**<0.05**	31.24±7.72	33.83±6.12	**<0.05**	**<0.05/<0.05**
Fat Mass (FATM), Kg	20.35±10.83	18.65±9.40	**<0.05**	19.84±10.25	19.04±9.34	NS	NS/**<0.05**
Muscle Mass (PMM), Kg	35.99±12.25	31.69±9.65	**<0.05**	39.22±12.68	33.31±9.41	**<0.05**	**<0.05/<0.05**
Free-Fat Mass (FFM), Kg	37.98±12.84	33.42±10.14	**<0.05**	41.37±13.30	35.12±9.90	**<0.05**	**<0.05/<0.05**
Total Body Water (TBW), %	27.79±9.36	24.46±7.43	**<0.05**	30.28±9.70	25.71±7.26	**<0.05**	**<0.05/<0.05**
Basal Metabolic Rate (BMR), Kj	6604.51±1334.04	5608.80±994.64	**<0.05**	6888.60±1353.53	5734.12±962.3	**<0.05**	**<0.05/<0.05**
Bone Mass, Kg	1.98±0.59	1.72±0.50	**<0.05**	2.14±0.61	1.80±0.48	**<0.05**	**<0.05/<0.05**

Abbreviations: ApoA1, apolipoprotein A1; ApoB, apolipoprotein B; DBP, diastolic blood pressure; FT_4, free thyroxine; HbA1C, glycated haemoglobin; HDL, high-density lipoprotein; HOMA-IR, homeostatic model assessment for insulin resistance; LDL, low-density lipoprotein; Lp(a), Lipoprotein (a); SBP, systolic blood pressure; T_3, triiodothyronine; TG, triglycerides; TSH, thyroid-stimulating hormone.
Variables are presented as means ± standard deviation (SD); p values between time points were derived by comparisons between the two assessments using Paired T test or Wilcoxon's signed rank test for skewed variables; statistically significant associations are shown in bold. Statistical significance at $p < 0.05$ (rounded to 0.05 in the Table), NS: non-significant ($p > 0.05$) difference.

Table S2: Cardiometabolic risk factors and thyroid hormones at initial and annual assessment: Comparisons according to pubertal status (irrespective of gender)

	Initial Assessment			Annual Assessment			
	**Pre-pubertal**	**Pubertal**	**P within**	**Pre-pubertal**	**Pubertal**	**P within**	**P between timepoints (pre/pubertal vs pubertal)**
SBP, mmHg	107.84±11.65	114.78±11.95	**<0.05**	106.08±11.07	113.34±10.98	**<0.05**	NS/NS
DBP, mmHg	64.87±10.10	67.55±10.45	**<0.05**	64.34±9.54	67.89±8.68	**<0.05**	NS/NS
Cholesterol, mg/dL	160.89±28.71	154.13±26.25	**<0.05**	160.50±24.76	153.10±25.29	**<0.05**	NS/**<0.05**
HDL, mg/dL	53.33±13.03	50.61±11.46	**<0.05**	57.76±13.94	53.80±12.77	**<0.05**	**<0.05/<0.05**
LDL, mg/dL	93.29±25.71	87.42±22.60	**<0.05**	89.31±22.30	83.80±21.87	**<0.05**	**<0.05/<0.05**
Lp(a), mg/dL	16.14±23.65	16.49±23.14	NS	15.49±23.51	18.44±26.50	NS	**<0.05/<0.05**
TG, mg/dL	73.46±48.04	82.86±54.34	**<0.05**	72.17±51.72	79.14±39.24	**<0.05**	NS/**<0.05**
ApoB, mg/dL	76.45±17.99	72.59±16.32	**<0.05**	75.53±15.90	71.98±15.69	**<0.05**	NS/**<0.05**
Apo A1, mg/dL	143.42±20.20	138.03±20.30	**<0.05**	146.85±22.06	137.80±20.88	**<0.05**	NS/NS
Glucose, mg/dL	79.45±10.11	81.60±8.18	**<0.05**	80.89±7.39	82.49±7.41	**<0.05**	**<0.05/<0.05**
Insulin, μUI/mL	13.43±36.86	18.77±11.48	**<0.05**	11.82±8,57	17.11±9.17	**<0.05**	**<0.05/<0.05**
HbA1c %	5.21±0.25	5.24±0.24	**<0.05**	5.19±0.24	5.20±0.24	NS	NS/**<0.05**

Homa- IR	3.18±18.29	3.82±2.53	**<0.05**	2.41±1.89	3.51±1.99	**<0.05**	**<0.05**/NS
TSH, μU/mL	2.96±1.36	2.92±1.53	NS	2.99±1.54	2.81±1.46	NS	NS/<**0.05**
FT4, ng/dL	1.15±0.14	1.08±0.14	**<0.05**	1.14±0.13	1.06±0.14	**<0.05**	**<0.05**/NS
T3, ng/dL	149.28±31.47	136.80±26.64	**<0.05**	143.21±27.68	132.67±29.03	**<0.05**	**<0.05/<0.05**
Fat Percentage (FATP), %	33.40±6.80	34.68±7.25	**<0.05**	32.23±6.99	33.24±6.83	NS	**<0.05/<0.05**
Fat Mass (FATM), Kg	15.68±7.80	24.23±10.70	**<0.05**	14.75±7.68	22.72±9.93	**<0.05**	**<0.05**/NS
Muscle Mass (PMM), Kg	27.65±7.45	41.26±10.22	**<0.05**	27.34±7.27	41.61±10.42	**<0.05**	**<0.05/0.05**
Free-Fat Mass (FFM), Kg	29.21±7.82	43.48±10.73	**<0.05**	28.88±7.65	43.84±10.94	**<0.05**	**<0.05/0.05**
Total Body Water (TBW), %	21.37±5.72	31.82±7.82	**<0.05**	21.14±5.60	32.09±7.98	**<0.05**	**<0.05/<0.05**
Basal Metabolic Rate (BMR), Kj	5510.22±906.71	6791.56±1281	**<0.05**	5451.66±875.78	6771.22±1294.96	**<0.05**	**<0.05/<0.05**
Bone Mass, Kg	1.54±0.38	2.22±0.51	**<0.05**	1.53±0.37	2.23±0.52	**<0.05**	**<0.05/<0.05**

Abbreviations: ApoA1, apolipoprotein A1; ApoB, apolipoprotein B; DBP, diastolic blood pressure; FT_4, free thyroxine; HbA1C, glycated haemoglobin; HDL, high-density lipoprotein; HOMA-IR, homeostatic model assessment for insulin resistance; LDL, low-density lipoprotein; Lp(a), Lipoprotein (a); SBP, systolic blood pressure; T_3, triiodothyronine; TG, triglycerides; TSH, thyroid-stimulating hormone.

Variables are presented as means ± standard deviation (SD); p values between time points were derived by comparisons between the two assessments using Paired T test or Wilcoxon's signed rank test for skewed variables; statistically significant associations are shown in bold. Statistical significance at $p < 0.05$ (rounded to 0.05 in the Table), NS: non-significant ($p > 0.05$) difference.
